# Supplementary material for: Extended Texture Analysis of Non-Enhanced Whole-Body MRI Image Data for Response Assessment in Multiple Myeloma Patients Undergoing Systemic Therapy
Source: Cancers (Basel). 2020 Mar 24;12(3):761. doi: 10.3390/cancers12030761 (PMC7140042; doi:10.3390/cancers12030761)
Supplement: Supplementary file 1 [file cancers-12-00761-s001.zip › 738617-suppl/cancers-738617-supplementary.pdf]

Classes	Feature abbreviation	Definition
First-order statistics<br><br>Describe the distribution of voxel intensities within the image region defined by the mask through commonly used and basic metrics.	original_firstorder_10Percentile	10th percentile
	original_firstorder_90Percentile	90th percentile
	original_firstorder_Energy	Energy is a measure of the magnitude of voxel values in an image. A larger value implies a greater sum of the squares of these values.
	original_firstorder_Entropy	Entropy specifies the uncertainty/randomness in the image values. It measures the average amount of information required to encode the image values.
	original_firstorder_InterquartileRange	25th and 75th percentile of the image array
	original_firstorder_Kurtosis	Kurtosis is a measure of the 'peakedness' of the distribution of values in the image ROI. A higher kurtosis implies that the mass of the distribution is concentrated towards the tail(s) rather than towards the mean. A lower kurtosis implies the reverse: that the mass of the distribution is concentrated towards a spike near the Mean value.
	original_firstorder_Maximum	maximum
	original_firstorder_Mean	mean
	original_firstorder_MeanAbsoluteDeviation	Mean Absolute Deviation is the mean distance of all intensity values from the Mean Value of the image array.
	original_firstorder_Median	median
	original_firstorder_Minimum	minimum
	original_firstorder_Range	The range of gray values in the ROI.

original_firstorder_RobustMeanAbsoluteDeviation	Robust Mean Absolute Deviation is the mean distance of all intensity values from the Mean Value calculated on the subset of image array with gray levels in between, or equal to the 10th and 90th percentile.
original_firstorder_RootMeanSquared	Root Mean Squared (RMS) is the square-root of the mean of all the squared intensity values. It is another measure of the magnitude of the image values.
original_firstorder_Skewness	Skewness measures the asymmetry of the distribution of values about the Mean value. Depending on where the tail is elongated and the mass of the distribution is concentrated, this value can be positive or negative.
original_firstorder_TotalEnergy	Total Energy is the value of Energy feature scaled by the volume of the voxel in cubic mm.
original_firstorder_Uniformity	Uniformity is a measure of the sum of the squares of each intensity value. This is a measure of the homogeneity of the image array, where a greater uniformity implies a greater homogeneity or a smaller range of discrete intensity values.
original_firstorder_Variance	Variance is the the mean of the squared distances of each intensity value from the Mean value. This is a measure of the spread of the distribution about the mean.
original_glcm_Autocorrelation	Autocorrelation is a measure of the magnitude of the fineness and coarseness of texture.

**Gray Level Co-occurrence Matrix**<br><br>Describes the second order joint probability function for the given region of co-occurring pixel intensities	original_glcm_ClusterProminence	Cluster Prominence is a measure of the skewness and asymmetry of the GLCM. A higher value implies more asymmetry about the mean while a lower value indicates a peak near the mean value and less variation about the mean.
	original_glcm_ClusterShade	Cluster Shade is a measure of the skewness and uniformity of the GLCM. A higher cluster shade implies greater asymmetry about the mean.
	original_glcm_ClusterTendency	Cluster Tendency is a measure of groupings of voxels with similar gray-level values.
	original_glcm_Contrast	Contrast is a measure of the local intensity variation, favoring values away from the diagonal. A larger value correlates with a greater disparity in intensity values among neighboring voxels.
	original_glcm_Correlation	Correlation is a value between 0 (uncorrelated) and 1 (perfectly correlated) showing the linear dependency of gray level values to their respective voxels in the GLCM.
	original_glcm_DifferenceAverage	Difference Average measures the relationship between occurrences of pairs with similar intensity values and occurrences of pairs with differing intensity values.
	original_glcm_DifferenceEntropy	Difference Entropy is a measure of the randomness/variability in neighborhood intensity value differences.

original_glcm_DifferenceVariance	Difference Variance is a measure of heterogeneity that places higher weights on differing intensity level pairs that deviate more from the mean.
original_glcm_Id	Inverse Difference (ID) (a.k.a. Homogeneity 1) is another measure of the local homogeneity of an image. With more uniform gray levels, the denominator will remain low, resulting in a higher overall value.
original_glcm_Idm	Inverse Difference Moment (IDM) (a.k.a Homogeneity 2) is a measure of the local homogeneity of an image. IDM weights are the inverse of the Contrast weights (decreasing exponentially from the diagonal i=j in the GLCM).
original_glcm_Idmn	Inverse Difference Moment Normalized (IDMN) IDMN (inverse difference moment normalized) is a measure of the local homogeneity of an image. IDMN weights are the inverse of the Contrast weights (decreasing exponentially from the diagonal i=j in the GLCM). Unlike Homogeneity2, IDMN normalizes the square of the difference between neighboring intensity values by dividing over the square of the total number of discrete intensity values.

original_glcm_Idn	Inverse Difference Normalized (IDN) is another measure of the local homogeneity of an image. Unlike Inverse Difference, IDN normalizes the difference between the neighboring intensity values by dividing over the total number of discrete intensity values.
original_glcm_Imc1	Informational Measure of Correlation (IMC) 1 assesses the correlation between the probability distributions of i and j (quantifying the complexity of the texture), using mutual information I(x, y).
original_glcm_Imc2	Informational Measure of Correlation (IMC) 2 also assesses the correlation between the probability distributions of i and j (quantifying the complexity of the texture).
original_glcm_InverseVariance	Inverse Variance of GLCM.
original_glcm_JointAverage	Joint average returns the mean gray level intensity of the i distribution.
original_glcm_JointEnergy	Energy is a measure of homogeneous patterns in the image. A greater Energy implies that there are more instances of intensity value pairs in the image that neighbor each other at higher frequencies.
original_glcm_JointEntropy	Joint entropy is a measure of the randomness/variability in neighborhood intensity values.
original_glcm_MaximumProbability	Maximum Probability is occurrences of the most predominant pair of neighboring intensity values.

	original_glcm_SumAverage	Sum Average measures the relationship between occurrences of pairs with lower intensity values and occurrences of pairs with higher intensity values.
	original_glcm_SumEntropy	Sum Entropy is a sum of neighborhood intensity value differences.
	original_glcm_SumSquares	Sum of Squares or Variance is a measure in the distribution of neigboring intensity level pairs about the mean intensity level in the GLCM.
**Gray level Dependence Matrix**<br><br>Quantifies gray level dependencies in an image. A gray level dependency is defined as a the number of connected voxels within distance δ that are dependent on the center voxel.	original_gldm_DependenceEntropy	Dependence Entropy (DE) measures the entropy in dependence size in the image.
	original_gldm_DependenceNonUniformity	Dependence Non-Uniformity (DN) measures the similarity of dependence throughout the image, with a lower value indicating more homogeneity among dependencies in the image.
	original_gldm_DependenceNonUniformityNormalized	Dependence Non-Uniformity Normalized (DNN) measures the similarity of dependence throughout the image, with a lower value indicating more homogeneity among dependencies in the image.
	original_gldm_DependenceVariance	Dependence Variance (DV) measures the variance in dependence size in the image.
	original_gldm_GrayLevelNonUniformity	Gray Level Non-Uniformity (GLN) measures the similarity of gray-level intensity values in the image, where a lower GLN value correlates with a greater similarity in intensity values.
	original_gldm_GrayLevelVariance	Gray Level Variance (GLV) measures the variance in grey level in the image.

original_gldm_HighGrayLevelEmphasis	High Gray Level Emphasis (HGLE) measures the distribution of the higher gray-level values, with a higher value indicating a greater concentration of high gray-level values in the image.
original_gldm_LargeDependenceEmphasis	Large Dependence Emphasis (LDE) is a measure of the distribution of large dependencies, with a greater value indicative of larger dependence and more homogeneous textures.
original_gldm_LargeDependenceHighGrayLevelEmphasis	Large Dependence High Gray Level Emphasis (LDHGLE) measures the joint distribution of large dependence with higher gray-level values.
original_gldm_LargeDependenceLowGrayLevelEmphasis	Large Dependence Low Gray Level Emphasis (LDLGLE) measures the joint distribution of large dependence with lower gray-level values.
original_gldm_LowGrayLevelEmphasis	Low Gray Level Emphasis (LGLE) measures the distribution of low gray-level values, with a higher value indicating a greater concentration of low gray-level values in the image.
original_gldm_SmallDependenceEmphasis	Small Dependence Emphasis (SDE) is a measure of the distribution of small dependencies, with a greater value indicative of smaller dependence and less homogeneous textures.

Gray Level Run Length Matrix

Quantifies gray level runs, which are defined as the length in number of pixels, of consecutive pixels that have the same gray level value.

original_gldm_SmallDependenceHighGrayLevelEmphasis	Small Dependence High Gray Level Emphasis (SDHGLE) measures the joint distribution of small dependence with higher gray-level values.
original_gldm_SmallDependenceLowGrayLevelEmphasis	
original_glrlm_GrayLevelNonUniformity	Gray Level Non-Uniformity (GLN) measures the similarity of gray-level intensity values in the image, where a lower GLN value correlates with a greater similarity in intensity values.
original_glrlm_GrayLevelNonUniformityNormalized	Gray Level Non-Uniformity Normalized (GLNN) measures the similarity of gray-level intensity values in the image, where a lower GLNN value correlates with a greater similarity in intensity values.
original_glrlm_GrayLevelVariance	Gray Level Variance (GLV) measures the variance in gray level intensity for the runs.
original_glrlm_HighGrayLevelRunEmphasis	High Gray Level Run Emphasis (HGLRE) measures the distribution of the higher gray-level values, with a higher value indicating a greater concentration of high gray-level values in the image.
original_glrlm_LongRunEmphasis	Long Run Emphasis (LRE) is a measure of the distribution of long run lengths, with a greater value indicative of longer run lengths and more coarse structural textures.
original_glrlm_LongRunHighGrayLevelEmphasis	Long Run High Gray Level Emphasis (LRHGLE) measures the joint distribution of long run lengths with higher gray-level values.
original_glrlm_LongRunLowGrayLevelEmphasis	Long Run Low Gray Level Emphasis (LRLGLE) measures the joint distribution of long run lengths with lower gray-level values.

original_glrlm_LowGrayLevelRunEmphasis	Low Gray Level Run Emphasis (LGLRE) measures the distribution of low gray-level values, with a higher value indicating a greater concentration of low gray-level values in the image.
original_glrlm_RunEntropy	Run Entropy (RE) measures the uncertainty/randomness in the distribution of run lengths and gray levels. A higher value indicates more heterogeneity in the texture patterns.
original_glrlm_RunLengthNonUniformity	Run Length Non-Uniformity (RLN) measures the similarity of run lengths throughout the image, with a lower value indicating more homogeneity among run lengths in the image.
original_glrlm_RunLengthNonUniformityNormalized	Run Length Non-Uniformity Normalized (RLNN) measures the similarity of run lengths throughout the image, with a lower value indicating more homogeneity among run lengths in the image.
original_glrlm_RunPercentage	Run Percentage (RP) measures the coarseness of the texture by taking the ratio of number of runs and number of voxels in the ROI.
original_glrlm_RunVariance	Run Variance (RV) is a measure of the variance in runs for the run lengths.
original_glrlm_ShortRunEmphasis	Short Run Emphasis (SRE) is a measure of the distribution of short run lengths, with a greater value indicative of shorter run lengths and more fine textural textures.
original_glrlm_ShortRunHighGrayLevelEmphasis	Short Run High Gray Level Emphasis (SRHGLE) measures the joint distribution of shorter run lengths with higher gray-level values.

	original_glrlm_ShortRunLowGrayLevelEmphasis	Short Run Low Gray Level Emphasis (SRLGLE) measures the joint distribution of shorter run lengths with lower gray-level values.
**Gray Level Size Zone (GLSZM)**<br><br>quantifies gray level zones in an image. A gray level zone is defined as a the number of connected voxels that share the same gray level intensity. A voxel is considered connected if the distance is 1 according to the infinity norm (26-connected region in a 3D, 8-connected region in 2D).	original_glszm_GrayLevelNonUniformity	Gray Level Non-Uniformity (GLN) measures the variability of gray-level intensity values in the image, with a lower value indicating more homogeneity in intensity values.
	original_glszm_GrayLevelNonUniformityNormalized	Gray Level Non-Uniformity Normalized (GLNN) measures the variability of gray-level intensity values in the image, with a lower value indicating a greater similarity in intensity values.
	original_glszm_GrayLevelVariance	Gray Level Variance (GLV) easures the variance in gray level intensities for the zones.
	original_glszm_HighGrayLevelZoneEmphasis	High Gray Level Zone Emphasis (HGLZE) measures the distribution of the higher gray-level values, with a higher value indicating a greater proportion of higher gray-level values and size zones in the image.
	original_glszm_LargeAreaEmphasis	Large Area Emphasis (LAE) is a measure of the distribution of large area size zones, with a greater value indicative of more larger size zones and more coarse textures.
	original_glszm_LargeAreaHighGrayLevelEmphasis	Large Area High Gray Level Emphasis (LAHGLE) measures the proportion in the image of the joint distribution of larger size zones with higher gray-level values.
	original_glszm_LargeAreaLowGrayLevelEmphasis	Large Area Low Gray Level Emphasis (LALGLE) measures the proportion in the image of the joint distribution of larger size zones with lower gray-level values.

original_glszm_LowGrayLevelZoneEmphasis	Low Gray Level Zone Emphasis (LGLZE) measures the distribution of lower gray-level size zones, with a higher value indicating a greater proportion of lower gray-level values and size zones in the image.
original_glszm_SizeZoneNonUniformity	Size-Zone Non-Uniformity (SZN) measures the variability of size zone volumes in the image, with a lower value indicating more homogeneity in size zone volumes.
original_glszm_SizeZoneNonUniformityNormalized	Size-Zone Non-Uniformity Normalized (SZNN) measures the variability of size zone volumes throughout the image, with a lower value indicating more homogeneity among zone size volumes in the image.
original_glszm_SmallAreaEmphasis	Small Area Emphasis (SAE) is a measure of the distribution of small size zones, with a greater value indicative of more smaller size zones and more fine textures.
original_glszm_SmallAreaHighGrayLevelEmphasis	Small Area High Gray Level Emphasis (SAHGLE) measures the proportion in the image of the joint distribution of smaller size zones with higher gray-level values.
original_glszm_SmallAreaLowGrayLevelEmphasis	Small Area Low Gray Level Emphasis (SALGLE) measures the proportion in the image of the joint distribution of smaller size zones with lower gray-level values.
original_glszm_ZoneEntropy	Zone Entropy (ZE) measures the uncertainty/randomness in the distribution of zone sizes and gray levels. A higher value indicates more heterogeneneity in the texture patterns.

	original_glszm_ZonePercentage	Zone Percentage (ZP) measures the coarseness of the texture by taking the ratio of number of zones and number of voxels in the ROI.
	original_glszm_ZoneVariance	Zone Variance (ZV) measures the variance in zone size volumes for the zones.
**Neighbouring Gray Tone Difference Matrix**<br>Quantifies the difference between a gray value and the average gray value of its neighbours within distance δ.	original_ngtdm_Busyness	Busyness is a measure of the change from a pixel to its neighbour. A high value for busyness indicates a ‘busy’ image, with rapid changes of intensity between pixels and its neighbourhood.
	original_ngtdm_Coarseness	Coarseness is a measure of average difference between the center voxel and its neighbourhood and is an indication of the spatial rate of change. A higher value indicates a lower spatial change rate and a locally more uniform texture.
	original_ngtdm_Complexity	Complexity: An image is considered complex when there are many primitive components in the image, i.e. the image is non-uniform and there are many rapid changes in gray level intensity.
	original_ngtdm_Contrast	Contrast is a measure of the spatial intensity change, but is also dependent on the overall gray level dynamic range. Contrast is high when both the dynamic range and the spatial change rate are high, i.e. an image with a large range of gray levels, with large changes between voxels and their neighbourhood.

original_ngtdm_Strength	Strenght is a measure of the primitives in an image. Its value is high when the primitives are easily defined and visible, i.e. an image with slow change in intensity but more large coarse differences in gray level intensities.
