# Supplementary material for: Extended Texture Analysis of Non-Enhanced Whole-Body MRI Image Data for Response Assessment in Multiple Myeloma Patients Undergoing Systemic Therapy
Source: Cancers (Basel). 2020 Mar 24;12(3):761. doi: 10.3390/cancers12030761 (PMC7140042; doi:10.3390/cancers12030761)
Supplement: Supplementary file 1 [file cancers-12-00761-s001.zip › 738617-suppl/cancers-738617suppl.docx]

supplementary material

MR-imaging protocol

Examinations were performed on a 1.5 T MR scanner (Magnetom Avanto, Siemens Healthcare) with high-gradient performance (amplitude, 40 mT/m; slew rate, 170 mT × m–1/ms). The MR scanner was equipped with a total imaging matrix system, which enables one to connect up to 76 surface coil elements and to use up to 18 of them for a given field of view (FOV). The patients were positioned supine and head-first in the magnet bore with five sets of phased-array surface coils installed simultaneously to cover the head, neck, chest and abdomen and pelvis to the upper thighs; the spine coils were embedded in the patient table.

A minimum of three MR-sequences (coronal T1-weighted spin-echo and fat-suppressed T2-weighted STIR sequences followed by whole-body single-shot spin-echo echo-planar DWI with a Stejskal-Tanner diffusion encoding scheme and spectral fat suppression) were measured in all patients. 2D T1-weighted turbo spin-echo images were obtained with the following parameters: TR/TE, 654/11; number of signals acquired (averages), 6; matrix, 192 × 256; FOV, 320 × 320 mm; and section thickness, 5 mm. STIR images were obtained with the following parameters: 7020/87; inversion time, 150 ms; echo-train length, 15; averages, 4; matrix, 192 × 256; FOV, 320 × 320 mm; and section thickness, 5 mm. The patients were instructed to breath regularly during the entire examination. Two trace b values, 50 and 800 s/mm^2^, were used. DWI was acquired in a transversal direction. Sequence parameters were 4300/60; averages, 2 with averaged three-scan trace; 100% sampling; flip angle = 90º; matrix, 117 × 192; FOV, 324 × 399 mm, section thickness, 4 mm; and no gap between slices. FOV was fixed to allow composing of multistep DWI and to generate 3D maximum-intensity-projection images. The resulting voxel size was 2.8 × 2.1 × 4 mm. Apparent diffusion coefficient (ADC) maps were automatically generated, taking into account both b values and assumption of a linear signal decay by the MR software. The lowest b value of 50 s/mm2 (instead of 0) was chosen to eliminate signals from vessels and to reduce perfusion effects whereas the highest b value (800 s/mm2) was set to enhance the lesion-to-normal tissue conspicuity. In all cases, the patients did breathe freely; however, the sequence is capable of triggering, e.g., for improved image quality for dedicated DWI of the liver. Spectral fat suppression was applied. Each slab consisted of 30 slices; measurement time for each slab was < 2′. Depending on the body length, total imaging time was in the range of 20–35 min. Automated shimming was performed for each step. Parallel imaging (generalized autocalibrating partially parallel acquisition) and a parallel acceleration factor of two were used to shorten TE and acquisition time and to reduce image distortion as a result of magnetic susceptibility.
